# Metropolitan Hospital Sunday Fund

**Published:** 1905-05-13

**Authors:** 


### METROPOLITAN HOSPITAL SUNDAY FUND.

A meeting of the Council of the Metropolitan Hospital Sunday Fund was held on Tuesday, the 9th inst., at the Mansion House, the Lord Mayor (Mr. Alderman John Pound), President and Treasurer, presiding.

Sir Edmund Hay Currie read the following letter from Mr. George Herring:—

1 Hamilton Place, Piccadilly, W.
2nd May, 1905.

Sir Edmund Hay Currie.

Dear Sir,—As on previous occasions, I will leave it to the ouncil of the Hospital Sunday Fund to choose whether to accept a cheque for £10,000 to the Fund, *or* an addition of 5s. to each £1 collected in places of worship on June 25th, my liability not to exceed £25,000.

I hope the calls on the charitable for building purposes will not decrease the collections for the *maintenance* of the hospitals, as I think that should be the primary object.

Yours truly,
George Herring.

Sir Edmund said this was the thirty-third year of the Fund and the seventh on which they had had the advantage of Mr. Herring's munificence. Last year his gift was about £12,000, and it rested wholly with the congregations to secure the full amount of £25,000, as it was a *sine qua non* that the one-fourth he offered would be given only on amounts contributed through places of worship. The appeals of some half-dozen large hospitals had concentrated the attention of the public on these charities, to the detriment of a large number of other most deserving hospitals, which are in great need of assistance towards the maintenance of their patients. The Sunday Fund gave only to the support of patients, and nothing for building purposes. There were 10,000 beds in the London Hospitals, and over 105,000 in-patients occupied them last year. The hospitals were showing great endeavours to reduce their expenditure, without impairing efficiency. The Fund would this year make grants to 220 hospitals and institutions on the recommendation of the Distribution Committee, the money collected being distributed in a few weeks. Last year they made grants to 211 institutions; surgical appliances to the number of 6,185; and 16,000 letters for special hospitals and convalescent homes were issued. The first year's collection, in 1873, amounted to £27,700; while last year it was £63,054. This year £100,000 was asked for, which would secure the full £25,000 from Mr. George Herring. In conclusion, Sir Edmund referred to the loss sustained by the Council owing to the death of Lord Stanhope, who had been connected with the Fund almost from its foundation, having been a member of the Council since 1874, and also of another very good friend, Sir Reginald Hanson, who had been a member of the Council for 20 years.

Sir Sydney Waterlow moved that the generous offer of Mr. George Herring to add 5s. to each £1 collected in places of worship on June 25th be accepted with the best thanks of the Council.

The motion was agreed to, and Mr. Deputy Wallace, representing the Corporation of the City of London, said he thought good would be done if the Lord Mayor called the attention of the mayors of the various metropolitan boroughs to the collection and get them to remind the ministers in their localities of it.

The Rev. C. H. Grundy suggested that this should be done by means of a letter which would, he thought, carry considerable weight.

The Lord Mayor promised that this should be done, and a vote of thanks on the motion of Archdeacon Sinclair, closed the proceedings.

